# Optimal lifestyle behaviors and 10‐year progression of arterial stiffness: The Multi‐Ethnic Study of Atherosclerosis

**DOI:** 10.1111/jch.14430

**Published:** 2022-02-08

**Authors:** Yacob G. Tedla, Adam Gepner, James H. Stein, Joseph A. Delaney, Chia‐Ying Liu, Philip Greenland

**Affiliations:** ^1^ Division of Epidemiology Department of Medicine Vanderbilt University Medical Center Madison Wisconsin USA; ^2^ Division of Cardiovascular Medicine University of Wisconsin Madison Madison Wisconsin USA; ^3^ College of Pharmacy University of Manitoba Winnipeg Canada; ^4^ Department of Radiology University of Wisconsin Madison Madison Wisconsin USA; ^5^ Department of Preventive Medicine Feinberg School of Medicine Northwestern University Boston USA

**Keywords:** alcohol, arterial stiffness, body mass index, exercise, healthy lifestyle, smoking

## Abstract

Majority of previous studies showed no association between a single health behavior and arterial stiffness, but the benefit of simultaneously having multiple healthy behaviors (optimal lifestyle) on the progression of arterial stiffness is unknown. Among 2810 individuals (age 60.0 ± 9.4, 46.5% male), optimal lifestyle marker (yes/no) on four health behaviors (ie, BMI < 25 kg/m^2^, never or former smoker, never or moderate drinker, exercised > 500 METS min/week) across four visits (≈ 5 years) were summed to create an optimal lifestyle score. Carotid arterial stiffness was measured using distensibility coefficient (DC) and Young's elastic modulus (YEM) at visit 1 and after a mean of 9.5 years (visit 5). The association of optimal lifestyle with 10‐year percent change in DC and YEM was assessed using multiple linear regression. DC decreased by 5.3% and YEM increased by 24.4% over 10 years. Mean optimal lifestyle score was 9.4 ± 3.1 (range: 0–16). Individuals in quintiles 2–5 of optimal lifestyle score compared to quintile 1 (with the least optimal lifestyle score) did not show slower deceleration of DC [Q2, −0.3% (95% CI: −6.0, 5.4); Q3, −0.01% (−4.5, 4.5); Q4, −0.6% (−5.2, 3.9); Q5, −0.4% (−5.3, 4.4)], trend *p*‐value = .82] or slower progression of YEM [Q2, 0.1% (−7.1, 7.3); Q3, −0.8% (−8.0, 6.5); Q4, 4.5% (−2.3, 11.3); Q5, −0.2% (−8.3, 7.9)], trend *p*‐value = .49] after adjusting for risk factors. The association remained non‐significant when stratified by categories of age, sex, race, BP control, and diabetes. Our findings indicate that optimal score on multiple health behaviors may not independently slow arterial stiffness progression.

## 1 INTRODUCTION

Arterial stiffness is caused by a loss of the capacity of elastic arteries to stretch and increase arterial diameter in response to changes in blood pressure (BP).^1^, ^2^ Greater arterial stiffness is an independent predictor of cardiovascular events, chronic kidney disease (CKD), and all‐cause mortality.^3^, ^4^, ^5^ Aging and elevated BP are major risk factors for arterial stiffness.^6^, ^7^, ^8^

Healthy lifestyle habits such as regularly exercising, avoiding tobacco smoking and alcoholic beverages, and maintaining optimal body weight were associated with anti‐oxidative and anti‐inflammatory effects, reduced pro‐inflammatory cytokines, and production of nitric oxide within vascular endothelium.^9^, ^10^, ^11^, ^12^, ^13^, ^14^, ^15^, ^16^, ^17^, ^18^, ^19^ These physiological signals may reduce structural changes within the arterial wall such as fragmentation of elastin, deposition of collagen, and smooth muscle proliferation which are precursors of arterial stiffness.^9^ Despite these favorable effects on the artery, individual health behavior have not been consistently associated with arterial stiffness.^20^, ^21^, ^22^, ^23^ In a systematic review of 77 studies^20^ and meta‐analysis of 41 randomized controlled trials,^21^ the majority of studies (≥ 80%) found no association of arterial stiffness with smoking, body mass index (BMI) and aerobic exercise. Whether simultaneously maintaining favorable scores on multiple health behaviors (optimal lifestyle) slow the progression of arterial stiffness is unknown.

The objective of this study was to investigate the association between long‐term (≈ 5 years) optimal lifestyle (ie, BMI < 25 kg/m^2^, never smoker or quit smoking ≥12 months ago, never or moderate drinker, and exercised > 500 METS –/week) and 10‐year progression of arterial stiffness.

## 2 MATERIALS AND METHODS

### 2.1 Study design and participants

The Multi‐Ethnic Study of Atherosclerosis (MESA) is a population based longitudinal study of risk factors for subclinical and clinical cardiovascular diseases (CVD).^24^ In the MESA, 6814 men and women aged 44–84 years and free of cardiovascular events were recruited between July 2000 and August 2002 (visit 1, baseline) from 6 centers across the United States (Baltimore, Maryland; Chicago, IL; Forsyth County, NC, USA; Los Angeles County, CA, USA; Northern Manhattan, NY, USA; and St. Paul, MN, USA). The study protocol was approved by the institutional review boards at each field center, and written informed consent was obtained from all participants. Follow up examinations were conducted between September 2002 and February 2004 (visit 2), March 2004 and September 2005 (visit 3), September 2005 and May 2007 (visit 4), and April 2010 and December 2011 (visit 5). Participants were included in the current analysis if they had ultrasonography of the common carotid artery at visits 1 and 5 and a non‐missing value for lifestyle factors and covariates.

### 2.2 Carotid artery stiffness

Ultrasound images of the right common carotid artery, approximately 1 cm below the carotid bulb, were obtained using a Logiq 700 ultrasound system (General Electric Medical Systems, transducer frequency 13 MHz) at visits1 and 5. Images were digitized at high resolution using a Medical Digital Recording device (PACSGEAR, Pleasanton, CA, USA). Immediately before obtaining ultrasound images, supine brachial BP was measured using a standardized protocol with an automated upper arm sphygmomanometer (DINAMAP, GE Medical Systems, Milwaukee, WI, USA) after participants rested for 10 min in supine position. These BP measurements were used to calculate brachial artery pulse pressure (ΔP) in the calculation of arterial stiffness indicators.

Carotid artery systolic and diastolic diameters were determined by the largest and smallest diameters during the cardiac cycle. Three measurements from three consecutive cardiac cycles were taken to derive mean internal diameter at peak systole (D_s)_ and end‐diastole (D_d_), and external (D_e_) diameters at end‐diastole. Ninety percent of the readings were performed by two different readers. Inter‐reader reliability was excellent with intra‐class correlation 0.998 for internal end‐diastolic diameter, 0.98 for vessel wall thickness, and 0.85 for delta diameter (peak systolic internal diameter minus internal end‐diastolic diameter).^25^ Intra‐reader reliability test was performed on 25 representative images. Reproducibility was excellent with intra‐class correlation of 0.99 for internal end‐diastolic diameter, 0.92 for vessel wall thickness, and 0.87 for delta diameter (peak systolic internal diameter minus internal end‐diastolic diameter).^25^ Using formulas recommended by expert consensus,^26^ carotid artery distensibility coefficient (DC) was calculated as (D_s_^2^‐D_d_^2^)/(ΔP*D^2^_d_) and Young's elastic modulus (YEM) as 3(1+(D^2^_d_/(D^2^_e_ − D^2^_d_))]/DC. Ten‐year percent changes in DC and YEM scores were calculated as [(visit5 – visit1)/|visit 1|]*100. DC and YEM are inversely related, and arterial stiffness corresponds to a higher score on YEM and a lower score on DC.

### 2.3 Optimal lifestyle

BMI was calculated as weight (measured by a calibrated scale to the nearest 0.5 kg) in kilograms divided by height (measured by a stadiometer to the nearest 0.1 cm) in meters squared and optimal BMI was defined as a score of < 25 kg/m^2^.^27^ Smoking status was classified as never (smoked < 100 cigarettes per lifetime), former (smoked ≥ 100 cigarettes, but stopped for ≥1 year prior to examination visit), or current smoker (smoked ≥ 100 cigarettes per lifetime and still smokes or quit < 1 years prior to examination visit). Participants who never smoked or quit smoking ≥12 months ago were considered as having optimal smoking status.^27^ Self‐reported frequency and duration of participation in nine different activities during a typical week in the past month were used to calculate total Metabolic Equivalent of Task (MET) minutes per week for moderate‐vigorous activities and a score of ≥500 MET min/week was considered optimal physical activity.^28^ Participants were asked, “Have you ever consumed alcoholic beverages?” “Do you presently drink alcoholic beverages?” and “number of drinks of drinks per week”. Optimal alcohol consumption was defined as never or moderate alcohol consumption (ie, ≤14 drinks/week for men and ≤7 drinks/week for women). Number of optimal lifestyle indicator (yes/no) on four health behaviors (ie, BMI < 25 kg/m^2^, never or former smoker, never or moderate drinker, exercised > 500 METS min/week) across visits 1–4 were summed to create an overall optimal lifestyle score (ranged 0–16).

### 2.4 Statistical analysis

Multiple imputation using chained equations with 50 repetitions was used to impute missing values on adjusted covariates. In a multiple linear regression, we evaluated the association between quintiles of overall optimal lifestyle score and 10‐year change in carotid artery DC and YEM using quintile 1 (least healthy) as a reference group. Trend tests were performed by including quintile of optimal lifestyle as a continuous ordinal variable. We also examined the association between optimal score of each individual health behavior across visits 1–4 and 10‐year change in DC and YEM using participants with optimal score of zero (ie, no optimal score for individual health behavior across all visits 1–4) as a reference group. Our analysis was weighted by inverse probability of being a participant in this study because individuals included in our analysis were younger and less likely to be hypertensive and diabetic compared to all eligible participants. Age, hypertension and diabetes are known to be strong predictors of arterial stiffness.^6^, ^7^, ^8^ In all the models, plots of the residuals against the fitted was checked to assess assumptions of linearity and homoscedasticity and to check outlier observations.^29^ All analyses were performed using Stata 16.1 (StataCorp. 2019).^30^

## 3 RESULTS

### 3.1 Participant characteristics

In the MESA, there were 6814 participants at baseline. Only a subset of MESA participants (ie, 2810) had ultrasonography imaging of the carotid artery at both visits 1 and 5 (see Figure S1–flow diagram of participants included in the analysis). Of those 2810, 24.6% (691 individuals) had missing value on covariates needed for adjustment and their values were imputed. There was no significant difference on most demographic and clinical characteristics, but participants include in our analysis (*n* = 2810) were on average slightly younger (60.0 vs. 62.2 years) and less likely to be hypertensive (42.9% vs. 48.5%) and diabetic (9.1% vs. 13.0%) compared to all eligible participants (*N* = 6814) (Table S1).

Participants’ age at baseline ranged from 44 to 84 years (mean age: 60.0 years), 47.2% were male, 38.5% were White, 27.8% were Black, 22.0% were Hispanic, and 11.8% were Chinese (Table 1). At baseline, individuals with the highest optimal lifestyle score (quintile 5) compared to those with the lowest score (quintile 1) were slightly older, less likely to be Blacks, Hispanic, current smokers, hypertensive, on antihypertensive medications but more likely to be Whites, Chinese, never smoker, former smokers, nondrinker or moderate drinker, exercisers and had lower BMI, systolic and diastolic BP, and eGFR.

**TABLE 1:** Baseline demographics and clinical characteristics by quintiles of optimal lifestyle score

		Quintiles of optimal score on four lifestyle factors across four visits					
	All participants	Quintile 1	Quintile 2	Quintile 3	Quintile 4	Quintile 5	
	(*N* = 2810, range, 0–16)	(range, 0–7)	(range, 8–8)	(range, 9–10)	(range, 11–12)	(range, 13–16)	Trend
Participant characteristics	Mean (SD)	Mean (SD)	Mean (SD)	Mean (SD)	Mean (SD)	Mean (SD)	*p*‐value
Age (years)	60.0 (9.4)	59.1 (9.2)	60.3 (9)	60.3 (6.5)	60.0 (10.9)	61.2 (10)	.002
Male (%)	46.5	40.8	44.0	47.0	52.1	50.9	<.001
Race (%)							
White	39.1	28.5	35.7	35.8	46.1	58.0	<.001
Black	26.2	32.5	29.9	26.6	23.9	12.5	<.001
Hispanic	20.8	30.1	23.4	20.8	13.2	11.7	<.001
Chinese	13.8	9.0	11.0	16.8	16.7	17.8	<.001
Smoking status (%)							
Never smoker	52.8	46.4	53.8	55.2	55.9	57.2	<.001
Former smoker	35.6	27.0	35.7	39.1	40.5	41.8	<.001
Current smoker	11.3	26.6	10.4	5.8	3.5	1.0	<.001
Moderate or nondrinker (%)	58.1	32.8	38.2	65.4	73.6	93.2	<.001
Exercise (met‐h/week)	27.5 (40.6)	14.3 (35.3)	28.5 (36.4)	26.2 (26.4)	37.6 (52.7)	39.2 (43.1)	<.001
BMI (kg/m^2^)	27.8 (5.0)	30.2 (4.8)	28.9 (4.7)	27.7 (3.2)	26.9 (5.5)	23.0 (1.8)	<.001
Systolic BP (mm Hg)	123.7 (20.1)	126.4 (20.4)	125.6 (19.3)	122.5 (13.4)	123.3 (23.6)	118.4 (20.3)	<.001
Diastolic BP (mm Hg)	718 (10.1)	72.2 (10.5)	72.7 (9.6)	71.0 (6.7)	72.3 (12.2)	69.9 (10.0)	.009
Hypertension (%)	42.9	48.8	46.7	42.2	41.6	30.0	<.001
HDL cholesterol (mg/dl)	51.6 (15.2)	48.9 (13.9)	50.6 (14.3)	51.1 (10)	52.4 (18.2)	57.5 (17.5)	<.001
Total Cholesterol (mg/dl)	194.1 (34.9)	193.6 (35.8)	192.2 (35.5)	196.5 (25.5)	194.2 (39.1)	193.7 (33)	.49
Diabetes mellitus (%)	9.1	12.6	11.5	8.1	7.9	2.9	<.001
GFR (ml/min/1.73m^2^)	79.5 (15.1)	81.7 (16.2)	79.9 (15.7)	78.5 (9.7)	78.2 (17.1)	78.1 (14.3)	<.001
Antihypertensive medication (%)	33.2	39.1	36.8	33.5	31.1	20.1	<.001
Lipid lowering medication (%)	15.0	15.1	15.7	16.5	15.6	10.7	.25

Abbreviations: BMI, body mass index; BP, blood pressure; GFR, glomerular filtration rate; HDL, high density lipoprotein; MET, Metabolic Equivalent of Task; SD, standard deviation.

Mean DC and YEM were 3.5 × 10^–3^ mm Hg^–1^ and 3.0 × 10^3^ mm Hg at baseline and changed to 3.0 × 10^–3^ mm Hg^–1^ and 3.4 × 10^3^ mm Hg at visit 5, respectively (Table 2). On average, DC decreased by 5.3% and YEM increased by 24.4% over 10 years. Mean optimal lifestyle score from all four health behaviors across visits 1–4 was 9.4 ± 3.1 (range: 0–16). Mean optimal score for each health behavior was 1.2 ± 1.7 for BMI, 2.7 ± 1.4 for exercise, 3.5 ± 1.2 for smoking, 1.9 ± 1.6 for drinking. The proportion of participants with optimal score on all four visits was 24.1% for BMI, 41.3% for exercise, 84.2% for smoking and 28.2% for drinking (Table S2).

**TABLE 2:** Baseline and year‐ten arterial stiffness indicators by categories of optimal lifestyle factors

	Optimal lifestyle score	Baseline DC	Year‐ten DC		Baseline YEM	Year‐ten YEM	
Exposure characteristics (*N* = 2810	Mean (SD)	Mean (SD)	Mean (SD)	DC Percent Change	Mean (SD)	Mean (SD)	YEM percent change
All participants	9.4 (3.1)	3.5 (1.5)	3.0 (1.4)	−5.3	3.0 (1.8)	3.4 (2.6)	24.4
Quintiles of optimal lifestyle							
Quintile 1 (range, 0–7)	5.6 (1.5)	3.3 (1.5)	2.9 (1.3)	−3.7	3.2 (2.2)	3.6 (2.6)	22.2
Quintile 2 (range, 8–8)	8.0 (0)	3.4 (1.5)	3.0 (1.4)	−3.4	3.1 (1.7)	3.4 (2.2)	22.5
Quintile 3 (range, 9–10)	9.5 (0.5)	3.5 (1.5)	3.1 (1.4)	−4.5	2.9 (1.6)	3.3 (2.5)	22.6
Quintile 4 (range, 11–12)	11.6 (0.5)	3.5 (1.5)	3.1 (1.5)	−6.0	2.9 (1.6)	3.5 (2.6)	28.9
Quintile 5 (range, 13–16)	14.4 (1.2)	4.0 (1.8)	3.3 (1.6)	−10.1	2.7 (1.3)	3.2 (3.0)	25.7

*Abbreviations*: BMI, body mass index; DC, distensibility coefficient; SD, standard deviation; YEM, Young's elastic modulus.

^*^Mean and SD values are (x10^–3^ mm Hg^−1^) for DC and (x10^3^ mm Hg) for YEM.

### 3.2 Optimal lifestyle and progression in arterial stiffness

**FIGURE 1:**
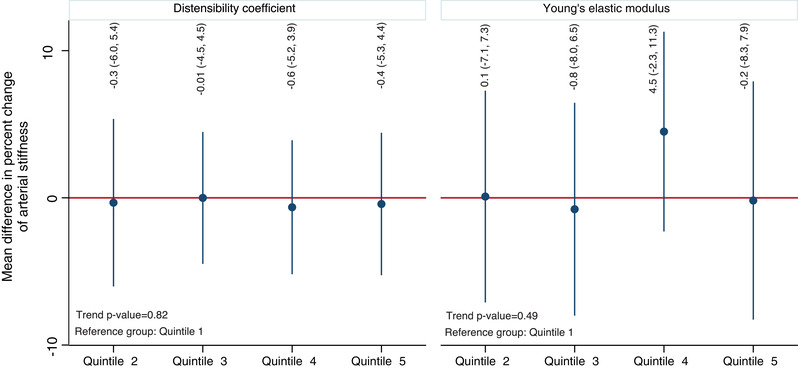
Linear regression association between quintiles of optimal lifestyle score (from four healthy behaviours ‐ BMI <25 kg/m^2^, never or former smoker, never or moderate drinker, exercised >500 METS min/wk) across four visits (≈5 years) and ten‐year percent change in distensibility coefficient (DC) and Young's elastic modules (YEM). Adjusted for baseline age, sex, race, study site, systolic and diastolic BP, diabetes mellitus, total cholesterol, HDL cholesterol, eGFR, anti‐hypertensive and lipid lowering medications, baseline DC or YEM, and change in systolic and diastolic BP between visits 1 and 4. Circular dots denote estimates and horizontal lines indicate the corresponding 95% confidence intervals

In models adjusted for age, sex, race, study site, baseline distensibility coefficient or Young's elastic modulus, there was no significant association between optimal lifestyle score and 10‐year arterial stiffness progression (Table S3: DC *p*‐trend = 0.66, YEM *p*‐trend = .96). After adjusting further for baseline systolic and diastolic BP, diabetes mellitus, total cholesterol, HDL cholesterol, glomerular filtration rate, use of antihypertensive and lipid medications, and change in BP between visits 1 and 4, individuals in quintiles 2–5 of optimal lifestyle score compared to quintile 1 (ie, those with the least optimal lifestyle score from the four health behaviors during all the four visits) did not show slower deceleration of DC [Q2, −0.3% (95% CI: −6.0, 5.4); Q3, −0.01% (−4.5, 4.5); Q4, −0.6% (−5.2, 3.9); Q5, −0.4% (−5.3, 4.4)], trend *p*‐value = .82] or slower progression of YEM [Q2, 0.1% (−7.1, 7.3); Q3, −0.8% (−8.0, 6.5); Q4, 4.5% (−2.3, 11.3); Q5, −0.2% (−8.3, 7.9)], trend *p*‐value = .49] (Figure 1). The association of optimal lifestyle score with percent change in DC and YEM was also not significant when stratified by age, sex, race, blood pressure control, and diabetes status (trend *p*‐value > = .05 for all) (Tables S4 and S5).

**FIGURE 2:**
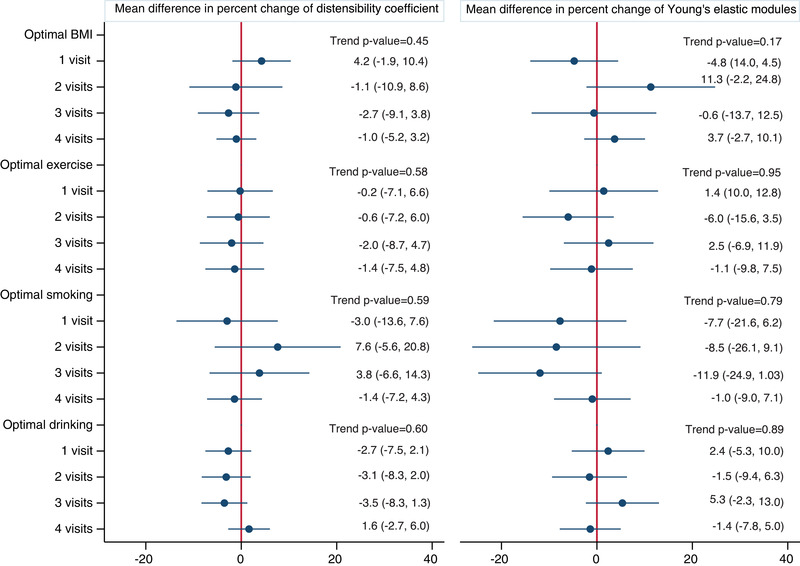
Linear regression association between number of visits with optimal health behaviours (BMI <25 kg/m^2^, never or former smoker, never or moderate drinker, exercised >500 METS min/wk) across four visits (≈ 5 years) and ten‐year percent change in distensibility coefficient and Young's elastic modules. Adjusted for baseline age, sex, race, study site, systolic and diastolic BP, diabetes mellitus, total cholesterol, HDL cholesterol, eGFR, anti‐hypertensive and lipid lowering medications, baseline DC or YEM, and change in systolic and diastolic BP between visits 1 and 4. Circular dots denote estimates and horizontal lines deonte 95% confidence intervals. Reference group are individuals with no optimal health behaviours at all visits

There was also no significant difference in the decline of DC or progression of YEM over 10 years by the individual health behaviors in the less adjusted model (Table S3: for BMI ‐ DC *p*‐trend = .88, YEM *p*‐trend = .52; for exercise ‐ DC *p*‐trend = .77, YEM *p*‐trend = .71; for smoking ‐ DC *p*‐trend = .63, YEM *p*‐trend = .75; and for drinking ‐ DC *p*‐trend = .34, YEM *p*‐trend = .54) and the fully adjusted model (Figure 2: for BMI ‐ DC *p*‐trend = .45, YEM *p*‐trend = .17; for exercise ‐ DC *p*‐trend = .58, YEM *p*‐trend = .95; for smoking ‐ DC *p*‐trend = .59, YEM *p*‐trend = .79; and for drinking ‐ DC *p*‐trend = .60, YEM *p*‐trend = .89).

## 4 DISCUSSION

In this study, using two measures of local arterial stiffness, we assessed whether long‐term optimal lifestyle on multiple health behaviors (BMI < 25 kg/m^2^, never or former smoker, never or moderate drinker, and exercise > 500 METS min/week) was associated with a slower progression in arterial stiffness over10‐years. Optimal score in individual health behaviors or simultaneous optimal score in all four health measures across four visits (≈ 5 years) was not associated with slower progression of arterial stiffness independent of age, systolic and diastolic BP, diabetes mellitus and other risk factors.

The absence of associations between each individual's health behavior and arterial stiffness concurs with findings from previous studies^20^, ^21^, ^22^, ^23^, ^31^, ^32^ In a systematic review study, Cecelja et al.^20^ investigated predictors of arterial stiffness from 77 studies that performed a multiple regression analysis to identify independent associations and concluded that the contribution of risk factors other than age and BP to arterial stiffness is small or insignificant. In Cecelja's study, smoking and BMI were not associated with arterial stiffness in ≥ 86% of the reviewed studies while age and BP were consistently associated in 91% and 90% of the studies, respectively.^20^ Likewise, a systematic review and meta‐analysis of 41 randomized controlled trials by Ashor et al.^21^ concluded no effect of a combination of aerobic and resistance exercise lasting ≥4 weeks on pulse wave velocity. Inconsistent association between alcohol consumption and arterial stiffness was also reported.^22^, ^23^, ^33^ While cross‐sectional studies reported J‐shaped association between alcohol intake and arterial stiffness,^23^, ^33^ prospective cohort study reported no association.^22^

Studies have shown that regularly exercising, not smoking and drinking, and maintaining optimal body weight have anti‐oxidative effects, increase anti‐inflammatory cytokines while reducing pro‐inflammatory cytokines, and enhances production of nitric oxide.^9^, ^10^, ^11^, ^12^, ^13^, ^14^, ^15^, ^16^, ^17^, ^18^, ^19^ These functional changes within vascular endothelium are believed to reduce the fragmentation of elastin, deposition of collagen, and smooth muscle proliferation which may result in slower progression for arterial stiffness.^9^ However, the findings of our study and other studies^21^, ^22^, ^23^, ^31^, ^32^, ^33^ indicate that the physiologic change in the vascular wall due to healthy lifestyle may not have significant impact on slowing arterial wall stiffening.

Unhealthy lifestyle is known to be a risk factor for elevated BP–a major modifiable risk factor for arterial stiffness. However, even in a model unadjusted for baseline BP and other risk factors (diabetes mellitus, total cholesterol, HDL cholesterol, glomerular filtration rate, use of antihypertensive and lipid medications), we did not find an association between healthy lifestyle and lesser progression in arterial stiffness (Table S3). Aging and uncontrolled BP are major risk factors for arterial stiffness,^6^, ^7^, ^8^ thus, we investigated if maintaining optimal lifestyle is associated with slower progression of arterial stiffness among younger or those with controlled BP. However, the association between optimal lifestyle and percent change in arterial stiffness remained non‐significant when stratified by different categories of age and blood pressure control (Tables S4 and S5).

Our study is the first, to our knowledge, to show maintaining healthy lifestyle simultaneously on multiple health behaviors is not independently associated with a slower progression in arterial stiffness. Some limitations should be noted. During a mean follow‐up time of 4.8 years, health behaviors were measured only at four visits, and this may not accurately reflect participants’ level of healthy lifestyle during that time. Brachial pulse pressure was used as a proxy for carotid pulse pressure in the calculation of DC and YEM. Brachial pulse pressure has been shown to overestimate central pulse pressure among younger individuals^26^, ^34^ and this may have over‐estimated arterial stiffness among younger participants. In addition, the first arterial stiffness measurement was made when individuals were about 10 years younger than the second measurement. Hence, 10‐year changes in arterial stiffness may have been underestimated, particularly in participants who transitioned from middle age to elderly between the two measurements. Furthermore, compared to all eligible participants, those included in our analysis were younger, less likely to have hypertension, diabetes and were healthier (more likely to be never smoker, nondrinkers or moderate drinkers, exercisers and had lower BMI). This may have limited generalizability of our findings, because if there is any beneficial effect of improving healthy lifestyles on arterial stiffness, the effect might be more noticeable in individuals with unhealthier lifestyles at baseline. However, our analysis was weighted by inverse probability of being a participant in this study to improve generalizability our findings. Underestimating smoking and drinking habits while overestimating exercise habit are also common^35^, ^36^, ^37^ and this non‐differential misclassification may have played a role in underestimating the true association between optimal lifestyle and arterial stiffness.

In conclusions, Prolonged (≈ 5 years) optimal lifestyle on multiple health behaviors (BMI < 25 kg/m^2^, never smoker or quit smoking ≥12 months ago, never drinker or current non‐drinker, and exercised > 500 METS min/week), although known to help at reducing risk of cardiac events,^38^, ^39^ was not associated with slower progression in arterial stiffness over 10‐years independent of age, systolic and diastolic BP, diabetes mellitus and other risk factors.

## CONFLICT OF INTEREST

None to disclose.

## AUTHOR CONTRIBUTIONS

Yacob G. Tedla conceptualized the study, performed data analysis, and drafted the manuscript. Adam Gepner, James H. Stein, and Philip Greenland were involved in the conceptualization of the study, data interpretation, and reviewed and edited the manuscript. Joseph A. Delaney and Chia‐Ying Liu reviewed and edited the manuscript and involved in data interpretation. All authors read and approved the final version of the manuscript.

## Supporting information

SUPPORTING INFORMATION

SUPPORTING INFORMATION
