# Supplementary material for: Optimal lifestyle behaviors and 10‐year progression of arterial stiffness: The Multi‐Ethnic Study of Atherosclerosis
Source: J Clin Hypertens (Greenwich). 2022 Feb 8;24(4):401–8. doi: 10.1111/jch.14430 (PMC8989754; doi:10.1111/jch.14430)
Supplement: Supplementary file 1 — SUPPORTING INFORMATION [file JCH-24-401-s001.docx]

Figure 1S: Flow diagram describing participants inclusion for analysis.

Readable ultrasound at both visits 1 and 5

n=2810 (analyzed)

Visit 1 and/or visit 5 readable ultrasound

n=2927

Visit 1 and visit 5 ultrasound taken n=3642

MESA participants at visit 1

N=6814

Note: Carotid artery ultrasound was taken at visits 1 and 5 to measure carotid artery stiffness.
