# Supplementary material for: Optimal lifestyle behaviors and 10‐year progression of arterial stiffness: The Multi‐Ethnic Study of Atherosclerosis
Source: J Clin Hypertens (Greenwich). 2022 Feb 8;24(4):401–8. doi: 10.1111/jch.14430 (PMC8989754; doi:10.1111/jch.14430)
Supplement: Supplementary file 2 — SUPPORTING INFORMATION [file JCH-24-401-s002.docx]

**Online-Only Supplemental**

| **Table 1s.** Baseline characteristics among eligible participants and participants included in the analysis | | | |
| --- | --- | --- | --- |
|  | Eligible participants | Participants included in analysis |  |
|  | (N=6,814) | (N=2,810) |  |
| Participant characteristics | Mean (SD) | Mean (SD) | *p*-value |
| Age (years) | 62.2(10.2) | 60.0(9.4) | <0.001 |
| Male (%) | 47.2 | 46.5 | 0.59 |
| Race (%) |  |  |  |
| White | 38.5 | 39.1 | 0.54 |
| Black | 27.8 | 26.2 | 0.12 |
| Hispanic | 22.0 | 20.8 | 0.20 |
| Chinese | 11.8 | 13.8 | 0.01 |
| Smoking status (%) |  |  |  |
| Never smoker | 50.2 | 52.8 | 0.01 |
| Former smoker | 36.5 | 35.6 | 0.51 |
| Current smoker | 13.0 | 11.3 | 0.02 |
| Moderate or nondrinker (%) | 55.9 | 58.1 | 0.05 |
| Exercise (met-hr/wk) | 25.9(39.0) | 27.5(40.6) | 0.06 |
| BMI (kg/m^2^) | 28.3(5.5) | 27.8(5.0) | <0.001 |
| Systolic BP (mm Hg) | 126.6(21.5) | 123.7(20.1) | <0.001 |
| Diastolic BP (mm Hg) | 71.9(10.3) | 718(10.1) | 0.50 |
| Hypertension (%) | 48.5 | 42.9 | <0.001 |
| HDL mg/dL) | 51.0(14.8) | 51.6(15.2) | 0.05 |
| Total Cholesterol (mg/dL) | 194.2(35.7) | 194.1(34.9) | 0.98 |
| Diabetes mellitus (%) | 13.0 | 9.1 | <0.001 |
| GFR (ml/min/1.73m^2^ ) | 78.0(16.3) | 79.5(15.1) | <0.001 |
| Antihypertensive medication (%) | 37.2 | 33.2 | <0.001 |
| Lipid lowering medication (%) | 16.2 | 15.0 | 0.14 |

| **Table S2.** Baseline, year-ten, and percent change of arterial stiffness indicators by number of visits with of optimal health behavior | | | | | | | |
| --- | --- | --- | --- | --- | --- | --- | --- |
| Exposure characteristics  (N=2,810) | Baseline DC | Year-ten DC | DC Percent  Change | Baseline YEM | Year-ten YEM | YEM Percent  Change |  |
|  | Mean (SD) | Mean (SD) |  | Mean (SD) | Mean (SD) |  |  |
| All participants | 3.5 (1.5) | 3.0 (1.4) | -5.3 | 3.0 (1.8) | 3.4 (2.6) | 24.4 |  |
| Optimal BMI (%) |  |  |  |  |  |  |  |
| 0 visits (61.0%) | 3.3 (1.4) | 2.9 (1.4) | -3 () | 3.1 (1.8) | 3.5 (2.4) | 21.8 () |  |
| 1 visit (6.3%) | 3.6 (1.6) | 3.3 (1.5) | -1.8 () | 2.9 (2.4) | 3.1 (2.7) | 19.4 () |  |
| 2 visits (4.1%) | 3.6 (1.6) | 3 (1.4) | -6.6 () | 3 (2) | 3.6 (2.3) | 36 () |  |
| 3 visits (4.5%) | 3.8 (1.7) | 3.1 (1.5) | -9.9 () | 2.7 (1.5) | 2.9 (2) | 20 () |  |
| 4 visits (24.1%) | 3.8 (1.7) | 3.2 (1.5) | -10.8 () | 2.8 (1.5) | 3.5 (3.1) | 31.4 () |  |
| Optimal exercise (%) |  |  |  |  |  |  |  |
| 0 visits (9.2%) | 3.3 (1.5) | 2.8 (1.3) | -2.8 () | 3.3 (2.4) | 3.6 (2.5) | 24.5 () |  |
| 1 visit (13.2%) | 3.4 (1.6) | 3 (1.3) | -3.5 () | 3.2 (2.3) | 3.6 (3.3) | 22.9 () |  |
| 2 visits (16.8%) | 3.4 (1.5) | 3 (1.4) | -4.4 () | 3 (1.6) | 3.3 (2) | 19.4 () |  |
| 3 visits (19.4%) | 3.5 (1.5) | 3 (1.5) | -6.4 () | 2.9 (1.6) | 3.5 (2.6) | 28.8 () |  |
| 4 visits (41.3%) | 3.6 (1.5) | 3.1 (1.5) | -6.2 () | 2.9 (1.6) | 3.4 (2.6) | 24.9 () |  |
| Optimal smoking (%) |  |  |  |  |  |  |  |
| 0 visits (8.1%) | 4.2 (1.7) | 3.5 (1.6) | -10.6 () | 2.6 (2.3) | 3 (2.4) | 27 () |  |
| 1 visit (2.3%) | 3.6 (1.4) | 3.2 (1.5) | -5.7 () | 2.9 (1.5) | 3.1 (1.6) | 16.5 () |  |
| 2 visits (1.8%) | 3.5 (1.4) | 3.4 (1.9) | 2.6 () | 3.1 (2.4) | 3.4 (2.7) | 22.4 () |  |
| 3 visits (3.6%) | 3.2 (1.5) | 2.9 (1.3) | -1.4 () | 3.3 (1.8) | 3.5 (2.7) | 16 () |  |
| 4 visits (84.2%) | 3.4 (1.5) | 3 (1.4) | -5.2 () | 3 (1.7) | 3.5 (2.6) | 25 () |  |
| Optimal alcohol intake (%) |  |  |  |  |  |  |  |
| 0 visits (31.6%) | 3.3 (1.5) | 2.8 (1.4) | -4.2 () | 3.3 (2.1) | 3.7 (2.9) | 24.7 () |  |
| 1 visit (16.0%) | 3.5 (1.5) | 3 (1.4) | -7.8 () | 2.9 (1.4) | 3.5 (2.4) | 28.7 () |  |
| 2 visits (10.3%) | 3.4(1.5) | 3.0(1.2) | -6.1 | 3.0(1.6) | 3.4(2.8) | 23.3 |  |
| 3 visits (14.0%) | 3.6 (1.6) | 3.1 (1.5) | -7.7 () | 2.9 (1.6) | 3.3 (1.8) | 27.2 () |  |
| 4 visits (28.2%) | 3.7 (1.6) | 3.3 (1.6) | -3.4 () | 2.8 (1.7) | 3.2 (2.6) | 20.6 () |  |
| Abbreviations: BMI, body mass index; DC, distensibility coefficient; SD, standard deviation; YEM, Young’s elastic modulus. *Mean and SD values are (x10^-3^ mm Hg-1) for DC and (x10^3^ mmHg) for YEM. | | | | | | | |

| **Table S3.** Linear regression association between optimal score on four lifestyle factors across 4 visits (≈5 years) and ten-year percent change in arterial stiffness indices. | | | | | |
| --- | --- | --- | --- | --- | --- |
|  | **Outcome: distensibility coefficient** | | **Outcome: Young’s elastic modulus** | | |
| N=2,810 | Coef. (95% CI) | p-value | Coef. (95% CI) | | p-value |
| Optimal BMI |  |  |  | | |
| No at all visits (ref) |  |  |  | |  |
| At 1 visit | 6.2 (0.3, 12) | 0.04 | -7.5 (-16.6, 1.7) | | 0.11 |
| At 2 visits | -0.4 (-10.2, 9.4) | 0.94 | 10.1 (-3.3, 23.4) | | 0.14 |
| At 3 visits | -1.6 (-7.9, 4.7) | 0.62 | -2.7 (-15.8, 10.3) | | 0.68 |
| At 4 visits | 0.2 (-3.7, 4.1) | 0.93 | 1.6 (-4.5, 7.6) | | 0.61 |
| Trend p-value |  | 0.88 |  | | 0.52 |
| Optimal exercise |  |  |  |  |  |
| No at all visits (ref) |  |  |  |  | |
| At 1 visit | -0.2 (-7.1, 6.7) | 0.95 | 1.3 (-10.3, 12.9) | 0.83 | |
| At 2 visits | -0.5 (-7.2, 6.2) | 0.89 | -6.5 (-16.4, 3.3) | 0.19 | |
| At 3 visits | -1.8 (-8.6, 4.9) | 0.59 | 2 (-7.6, 11.6) | 0.68 | |
| At 4 visits | -0.9 (-7.2, 5.3) | 0.77 | -2.2 (-11.1, 6.7) | 0.63 | |
| Trend p-value |  | 0.77 |  | 0.71 | |
| Optimal smoking |  |  |  |  | |
| No at all visits (ref) |  |  |  |  | |
| At 1 visit | -2.2 (-12.5, 8.2) | 0.68 | -7.9 (-21.4, 5.6) | 0.25 | |
| At 2 visits | 9.4 (-3.9, 22.6) | 0.17 | -10.8 (-28.7, 7.1) | 0.24 | |
| At 3 visits | 5.2 (-5.1, 15.4) | 0.32 | -13.2 (-26, -0.5) | 0.04 | |
| At 4 visits | -1 (-6.8, 4.7) | 0.73 | -1 (-8.9, 7) | 0.81 | |
| Trend p-value |  | 0.63 |  | 0.75 | |
| Optimal alcohol drinking |  |  |  |  | |
| No at all visits (ref) |  |  |  |  | |
| At 1 visit | -2.9 (-7.7, 2) | 0.25 | 2.3 (-5.3, 9.9) | 0.54 | |
| At 2 visits | -2.8 (-8, 2.4) | 0.29 | -2 (-9.9, 5.9) | 0.62 | |
| At 3 visits | -3.5 (-8.4, 1.3) | 0.15 | 5.2 (-2.6, 13) | 0.19 | |
| At 4 visits | 2.6 (-1.8, 7.0) | 0.25 | -3 (-9.4, 3.4) | 0.37 | |
| Trend p-value |  | 0.34 |  | 0.54 | |
| Optimal lifestyle score |  |  |  |  | |
| Quintile 1 (ref) |  |  |  |  | |
| Quintile 2 | 0.7 (-5.1, 6.4) | 0.82 | -1.4 (-8.7, 5.8) | 0.70 | |
| Quintile 3 | 1.0 (-3.5, 5.4) | 0.67 | -2.3 (-9.5, 5.0) | 0.54 | |
| Quintile 4 | 0.1 (-4.4, 4.8) | 0.96 | 3.2 (-3.6, 10.1) | 0.36 | |
| Quintile 5 | 1.7 (-2.9, 6.3) | 0.47 | -3.5 (-11.4, 4.3) | 0.37 | |
| Trend p-value |  | 0.66 |  | 0.96 | |
| Models adjusted for baseline age, sex, race, study site, baseline distensibility coefficient or Young’s elastic modulus. | | | | | |

| **Table S4.** Linear regression association between optimal score on four lifestyle factors across 4 visits (≈5 5 years) and ten-year percent change in arterial stiffness indices by age, sex, and race groups. | | | | | | | | | | | |
| --- | --- | --- | --- | --- | --- | --- | --- | --- | --- | --- | --- |
|  | Age | | | |  | Sex | | Race | | | |
|  | Age <50  (n=414) | | Age 50-64  (n=1414) | Age 65-74  (n=776) | Age ≥75  (n=206) | Male  (n=1308) | Female  (n=1502) | White  (n=1100) | Black  (n=736) | Hispanic  (n=586) | Asian  (n=388) |
| N=2,,810 | Coef. (95% CI) | | Coef. (95% CI) | Coef. (95% CI) | Coef. (95% CI) | Coef. (95% CI) | Coef. (95% CI) | Coef. (95% CI) | Coef. (95% CI) | Coef. (95% CI) | Coef. (95% CI) |
| **Outcome: distensibility coefficient** | |  |  |  |  |  |  |  |  |  |  |
| Optimal lifestyle score quartile 1 (ref) |  | |  |  |  |  |  |  |  |  |  |
| Optimal lifestyle score quartile 2 | 0.1 (-9.7, 9.9) | | 3.8 (-2.8, 10.5) | -7.5 (-17.0, 1.9) | 1.1 (-18.6, 20.8) | 2.7 (-2.9, 8.3) | -3.8 (-10.7, 3.2) | 0.3 (-6.1, 6.8) | -1.1 (-10.3, 8.2) | -2.6 (-12.5, 7.3) | -6.7 (-21, 7.6) |
| Optimal lifestyle score quartile 3 | 1.1 (-7.3, 9.5) | | 1.4 (-4.1, 6.9) | -8.7 (-18.4, 1.0) | -13.6 (-35.3, 8.2) | 2.4 (-3.3, 8.0) | -4.5 (-11.6, 2.7) | 1.0 (-4.6, 6.6) | -0.5 (-9.1, 8.0) | -2.2 (-13.9, 9.4) | -2.8 (-18.9, 13.4) |
| Optimal lifestyle score quartile 4 | 5.2 (-5.2, 15.6) | | 2.5 (-4.1, 9.1) | -9.1 (-19.7, 1.5) | -10.3 (-32.2, 11.7) | 1.6 (-3.9, 7.1) | -4.1 (-11.6, 3.5) | -0.6 (-7.0, 5.8) | -3.7 (-13.6, 6.2) | 2.1 (-11.3, 15.6) | 0 (-15.5, 15.6) |
| Trend p-value | 0.41 | | 0.6 | 0.08 | 0.19 | 0.58 | 0.26 | 0.99 | 0.55 | 0.81 | 0.97 |
| **Outcome: Young’s elastic modulus** |  | |  |  |  |  |  |  |  |  |  |
| Optimal lifestyle score quartile 1 (ref) |  | |  |  |  |  |  |  |  |  |  |
| Optimal lifestyle score quartile 2 | -0.7 (-13.3, 12.0) | | -5.3 (-14, 3.5) | 12.9 (-3.8, 29.5) | -0.3 (-33.6, 32.9) | -4.9 (-14.5, 4.7) | 5.0 (-3.7, 13.8) | 2.7 (-7.8, 13.2) | -4.0 (-17.8, 9.7) | -0.04 (-13.4, 13.3) | -0.1 (-24.4, 24.2) |
| Optimal lifestyle score quartile 3 | 2.7 (-9.5, 14.8) | | -1.3 (-9.4, 6.7) | 5.7 (-7.3, 18.6) | 68.1 (9.9, 126.3) | -3.0 (-11.6, 5.7) | 11.3 (1.5, 21) | 1.4 (-7.0, 9.8) | -8.1 (-20.6, 4.4) | 5.6 (-10.2, 21.4) | 8.4 (-10.9, 27.8) |
| Optimal lifestyle score quartile 4 | -0.9 (-17.4, 15.7) | | -4.9 (-15.2, 5.5) | 10.9 (-5.8, 27.7) | -4.8 (-56.9, 47.3) | -3.0 (-12.8, 6.7) | 0.3 (-11.4, 12) | 5.1 (-6.4, 16.6) | 5.7 (-11.5, 22.9) | 1.1 (-16, 18.3) | -9 (-26.7, 8.8) |
| Trend p-value | 0.87 | | 0.56 | 0.42 | 0.53 | 0.62 | 0.39 | 0.44 | 0.8 | 0.66 | 0.76 |
| Adjusted for baseline age, sex, race, study site, systolic and diastolic BP, diabetes mellitus, total cholesterol, HDL cholesterol, eGFR, anti-hypertensive and lipid lowering medications, baseline DC or YEM, and change in systolic and diastolic BP between visits 1 and 4. | | | | | | | | | | | |

| **Table S5.** Linear regression association between optimal score on four lifestyle factors across 4 visits (≈5 5 years) and ten-year percent change in arterial stiffness indices by blood pressure control and diabetes status. | | | | | | | |
| --- | --- | --- | --- | --- | --- | --- | --- |
| Blood pressure control | | | | | | Diabetes mellitus | |
|  | BP control  at 0-1 visit  (n=308) | | BP control  at 2 visits  (n=288) | BP control  at 3 visits  (n=493) | BP control  at 4 visits  (n=1721) | Non-diabetic  (n=2561) | Diabetic  (n=249) |
| N=2,119 | Coef. (95% CI) | | Coef. (95% CI) | Coef. (95% CI) | Coef. (95% CI) | Coef. (95% CI) | Coef. (95% CI) |
| **Outcome: distensibility coefficient** | |  |  |  |  |  |  |
| Optimal lifestyle score quartile 1 (ref) |  | |  |  |  |  |  |
| Optimal lifestyle score quartile 2 | 3.6 (-9.6, 16.8) | | -5.6 (-24.1, 12.9) | -2 (-12.3, 8.2) | 0.7 (-4.5, 5.9) | 0.5 (-4.4, 5.5) | -3.3 (-18.2, 11.7) |
| Optimal lifestyle score quartile 3 | 8.2 (-7.1, 23.6) | | -2.4 (-18.9, 14.1) | -3.6 (-14, 6.9) | -0.7 (-6.3, 4.8) | -0.1 (-4.3, 4.1) | -14.5 (-32.9, 4) |
| Optimal lifestyle score quartile 4 | 0 (-14.2, 14.2) | | -14 (-31.5, 3.6) | -2.7 (-13.1, 7.6) | -0.1 (-6.1, 6) | 0.1 (-4.9, 5) | -18.3 (-37.5, 0.9) |
| Trend p-value | 0.83 | | 0.17 | 0.55 | 0.82 | 0.95 | 0.04 |
| **Outcome: Young’s elastic modulus** |  | |  |  |  |  |  |
| Optimal lifestyle score quartile 1 (ref) |  | |  |  |  |  |  |
| Optimal lifestyle score quartile 2 | -2.2 (-28, 23.5) | | 9.3 (-10.2, 28.7) | 5.6 (-8.3, 19.5) | -4.2 (-11.7, 3.2) | -0.1 (-7.2, 7) | -17.4 (-52.5, 17.8) |
| Optimal lifestyle score quartile 3 | 8.9 (-20.5, 38.3) | | -5.3 (-23.9, 13.2) | 5.1 (-9.3, 19.5) | 2.5 (-5.8, 10.8) | 2.6 (-3.5, 8.7) | 8.3 (-26.4, 43) |
| Optimal lifestyle score quartile 4 | 3.9 (-35.8, 43.7) | | 8.7 (-10.3, 27.7) | 7.1 (-10.8, 25) | -2.4 (-12.6, 7.8) | -1.5 (-9.6, 6.7) | 18.2 (-17.8, 54.3) |
| Trend p-value | 0.73 | | 0.62 | 0.43 | 0.86 | 0.85 | 0.22 |

**Additional covariates measurement**

After participants rested for 5 minutes in a quiet environment, 3 seated brachial BP measurements were taken by a trained and certified research staff in the right arm at an interval of 1 minute using a calibrated Dinamap PRO 100 automated oscillometric device (Critikon, Tampa, FL)1 with the back and arm supported during each visit. The last 2 measurements were used to calculate averaged BP at each visit.

Blood samples were obtained after 12-hours of fasting, and total cholesterol and high-density lipoprotein (HDL-C) were measured using cholesterol oxidase method (Roche Diagnostics, Indianapolis, Indiana) and serum glucose was measured using glucose oxidase method (Johnson & Johnson Clinical Diagnostics Inc., Rochester, NY). Creatinine amidinohydrolase method (Johnson & Johnson Clinical Diagnostics Inc) was used to measure serum creatinine and Glomerular filtration rate (GFR ml/min/1.73m^2^) was estimated using the Modification of Diet in Renal Disease equation.^2^ Diabetes mellitus was defined as having fasting blood glucose ≥126 mg/dL or taking antidiabetic medications.

**Reference**

[1] Critikon Dinamap Pro—Integrated Medical Systems. DINAMAP PRO Series 100–400 Monitor Operation Manual. Tampa, FL, 2002.

[2] Stevens LA, Coresh J, Feldman HI, Greene T, Lash JP, Nelson RG, Rahman M, Deysher AE, Zhang YL, Schmid CH, Levey AS. Evaluation of the modification of diet in renal disease study equation in a large diverse population. J Am Soc Nephrol. 2007;18:2749-2757.

**Author contributions**

YGT conceptualized the study, performed data analysis, and drafted the manuscript. AG, JHS, and PG were involved in the conceptualization of the study, data interpretation, and reviewed and edited the manuscript. JAD and CYL reviewed and edited the manuscript and involved in data interpretation. All authors read and approved the final version of the manuscript.
